# Independent, additive and interactive effects of acute normobaric hypoxia and cold on submaximal and maximal endurance exercise

**DOI:** 10.1007/s00421-023-05343-9

**Published:** 2023-11-14

**Authors:** A. Callovini, A. Fornasiero, A. Savoldelli, M. Decet, S. Skafidas, B. Pellegrini, L. Bortolan, F. Schena

**Affiliations:** 1 CeRiSM, Sport Mountain and Health Research Centre, University of Verona, Rovereto, Italy; 2 Department of Neurosciences, Biomedicine and Movement Sciences, University of Verona, Verona, Italy; 3 Present Address: Department of Cellular, Computational and Integrative Biology, University of Trento, Trento, Italy; 4 Present Address: Department of Engineering for Innovation Medicine, University of Verona, Verona, Italy

**Keywords:** Cold, Hypoxia, Exercise, Lactate threshold, Endurance, Performance

## Abstract

**Purpose:**

To evaluate the independent and combined effects of hypoxia (FiO2 = 13.5%) and cold (− 20 °C) on physiological and perceptual responses to endurance exercise.

**Methods:**

14 trained male subjects ($\mathop {\text{V}}\limits^{.}$O_2max_: 64 ± 5 mL/kg/min) randomly performed a discontinuous maximal incremental test to exhaustion on a motorized treadmill under four environmental conditions: Normothermic-Normoxia (N), Normothermic-Hypoxia (H), Cold-Normoxia (C) and Cold-Hypoxia (CH). Performance and physiological and perceptual responses throughout exercise were evaluated.

**Results:**

Maximal WorkLoad (WL) and WL at lactate threshold (LT) were reduced in C (− 2.3% and − 3.5%) and H (− 18.0% and − 21.7%) compared to N, with no interactive (p = 0.25 and 0.81) but additive effect in CH (− 21.5% and − 24.6%). Similarly, HR_max_ and Ve_max_ were reduced in C (− 3.2% and − 14.6%) and H (− 5.0% and − 7%), showing additive effects in CH (− 7.7% and − 16.6%). At LT, additive effect of C (− 2.8%) and H (− 3.8%) on HR reduction in CH (− 5.7%) was maintained, whereas an interactive effect (p = 0.007) of the two stressors combined was noted on Ve (C: − 3.1%, H: + 5.5%, CH: − 10.9%). [La] curve shifted on the left in CH, displaying an interaction effect between the 2 stressors on this parameter. Finally, RPE at LT was exclusively reduced by hypoxia (*p* < 0.001), whereas TS_max_ is synergistically reduced by cold and hypoxia (interaction *p* = 0.047).

**Conclusion:**

If compared to single stress exposure, exercise performance and physiological and perceptual variables undergo additive or synergistic effects when cold and hypoxia are combined. These results provide new insight into human physiological responses to extreme environments.

## Introduction

Real-world extreme environments often combine multiple environmental stressors, thereby making their overall effects on the individuals less predictable. High altitude is characterized by this ‘extreme’ nature, as it often displays the simultaneous presence of numerous stressful factors (e.g., hypobaric hypoxia and cold) (Lloyd and Havenith 2016). However, despite the independent effect of hypoxia (Fulco et al. 1998; Robert S.Mazzeo 2006) and cold (Castellani and Tipton 2016; Oksa et al. 2004; Stensrud et al. 2007; Taylor et al. 2008; No and Kwak 2016) has been well studied in literature, scarce knowledge is present on the combined effect of the two environmental conditions on human physiology and performance, especially when considering endurance exercise (Mugele et al. 2021; Bortolan et al. 2021). In fact, currently only 2 studies (Lloyd et al. 2015, 2016) have examined the individual and combined effects of cold and hypoxia on performance at altitude, but none of them investigated physiological and mechanical work responses during whole body dynamic endurance exercises like cycling or running. To date research on single stress exposure suggests that many of the key physiological strains associated with thermal cold and hypoxia are precursors of detrimental effects on exercise capacity; it is well known that, as altitude increases, the systemic reduction in arterial O_2_ content strains the cardiovascular system’s ability to meet the required O_2_ delivery to active musculature (Amann et al. 2006; Fulco et al. 1998), causing a linear decrease in maximal oxygen uptake ($\mathop {\text{V}}\limits^{.}$O_2max_) corresponding to ≈6.3% per 1000 m increasing altitude in endurance trained athletes up to 3000 m (Wehrlin and Hallén, 2006). However, despite great differences in relative exercise intensity, submaximal oxygen uptake at a specific external workload is similar at sea level and altitude (Fulco et al. 1998; Wehrlin and Hallén, 2006). Higher controversy exists on $\mathop {\text{V}}\limits^{.}$O_2max_ changes in the cold: Oksa et al. (2004) and Quirion et al. (1989) reported a 5% decrease in $\mathop {\text{V}}\limits^{.}$O_2max_ at − 20 °C if compared to + 20 °C, whereas Renberg et al. (2014) and Sandsund et al. (2012) claim no changes in ambient temperatures between − 14 and + 20, and Therminaris et al. (1989) found a 13% increase in $\mathop {\text{V}}\limits^{.}$O_2peak_ at −2 °C if compared to + 24 °C. These results suggest that V̇O_2max_ values may be affected in the cold for ambient temperatures lower than −15 °C, and the proposed reason for this decrease are the cold-induced local vasoconstriction that reduces venous washout of metabolic by-products in the active muscles (Oksa et al. 2004; Quirion et al. 1989), reduced ventilation due to cold-induced bronchus constriction (Kennedy et al. 2019) or cooling-induced neuromuscular changes like decreased maximal force production or slower nerve conduction and muscle contraction velocity (Oksa 2002). More agreement exists in relation to higher $\mathop {\text{V}}\limits^{.}$O_2_ at submaximal exercise intensities in the cold (Oksa et al. 2004; Quirion et al. 1989; Therminarias 1992; Therminarias et al. 1989) due to both a reduction in the mechanical efficiency of working muscles and to the shivering produced by muscles not involved in muscular exercise(Oksa 2002; Therminarias 1992).

As $\mathop {\text{V}}\limits^{.}$O_2max_, also aerobic performance is consequently affected by environmental condition. The state of art regarding maximal incremental test in hypoxia shows a 10 to 13% decrease in peak power output (PPO) or maximal aerobic velocity (VAM) at altitudes between 2500 and 3500 m if compared to sea level (Faulhaber et al. 2021; Friedmann et al. 2004, 2005; Lorenz et al. 2006; Ofner et al. 2014; Weckbach et al. 2019), and the same happens at the intensities associated with the lactate thresholds, with a reduction ranging from 12 to 19% considering different detecting methods (Faulhaber et al. 2021; Weckbach et al. 2019). Similarly, Quirion et al. (1989) found a 22% reduction in maximal WorkLoad (WL) and Oksa et al. (2004) a 9% decrease in running performance time when exposed to − 20 °C if compared to + 20 °C. Concerning WL at Lactate Threshold (LT), the same distinction between moderate (> − 15 °C) and severe (< −15 °C) cold previously mentioned for $\mathop {\text{V}}\limits^{.}$O_2max_ should be considered: in fact, Morrissey et al. (2019) found a 22% higher WL and Sandsund et al. (2012) a 10% increase in running speed at LT within − 4 and 1 °C if compared to 20 °C (suggesting this as the optimal ambient temperature range for aerobic endurance performance), whereas Renberg and colleagues (2014) found no differences in PO at − 14 °C if compared to + 20 °C in women. However, no information on mechanical work variation at LT when exposed to severe cold (i.e., −20 °C) is available.

Both $\mathop {\text{V}}\limits^{.}$O_2max_ and consequent aerobic performance reductions are linked to environmental induced changes in physiological responses to exercise, although the magnitude and mechanism of action for these changes are in many cases still unclear. For the purposes of this study, only responses related to acute environmental stressor exposure will be considered. HR_max_ has been shown to be reduced (Fornasiero et al. 2018; Grataloup et al. 2007; Mourot 2018; Ofner et al. 2014) when acutely exposed to hypoxic environments, the magnitude of this reduction being better explained by the altitude gain between normoxic and hypoxic incremental tests rather than by absolute altitude per se (i.e., 1.7 bpm per 1000 m gain in altitude (Garvican–Lewis et al. 2015), which corresponds to ≈3/4% reduction in HR_max_ for altitudes of 3500 m asl (Fornasiero et al. 2018; Ofner et al. 2014)). Changes in cardiac electrophysiological properties (Benoit et al. 2003; Mourot 2018) and a reduced central drive on the heart as a protective mechanism from myocardial ischemia (Noakes et al. 2001) have been addressed as possible mechanisms for HR_max_ reductions with acute hypoxic exposure. At submaximal exercise intensities, for the same external workload, HR in hypoxia is increased to meet exercising muscles oxygen requests (Clark et al. 2007). However, when considering workload in relative terms, HR in normoxia and hypoxia is similar (Ofner et al. 2014): this may explain why, despite absolute HR at lactate threshold seems to be reduced in hypoxia, when it is expressed as a percentage of maximal values in the respective conditions it shows no differences from sea level values (Friedmann et al. 2004, 2005). In the cold, HR_max_ reduction has been addressed as primarily responsible for the reduced $\mathop {\text{V}}\limits^{.}$O_2max_, decreasing from 10 to 30 bpm when deep body temperature is lowered by 0.5 to 2.0 °C (Castellani and Tipton 2016). Specifically, a percentage decrease ranging from − 2.5 to − 5.5% has been found for ambient temperatures between − 14 and − 20 °C if compared to + 20 °C (Oksa et al. 2004; Renberg et al. 2014; Sandsund et al. 2012). Submaximal HR changes in the cold is more controversial, with some studies showing a reduction (Sandsund et al. 2012) and other no changes (Renberg et al. 2014) for ambient temperatures lower than − 14 °C if compared to thermoneutral conditions. Cold induced peripheral vasoconstriction that results in an elevation of blood pressure, increased central blood volume and higher stroke volume (Doubt 1991; Gisolfi and Wenger 1984) seems to be responsible for a parasympathetically mediated reduction in HR (Doubt 1991; Sandsund et al. 2012; Taylor et al. 2008).

The lactate-power output/velocity curve is left shifted in hypoxia (Clark et al. 2007; Friedmann et al. 2005; Ofner et al. 2014), testifying greater reliance on anaerobic metabolism when comparing a same absolute exercise intensity. However, Ofner et al. (2014) found completely the same pattern of the curve and no significant difference in lactate concentration between normoxia and hypoxia in relative terms (i.e., same lactate concentration per watt in both environments). Furthermore, despite anaerobic threshold concepts are very popular to prescribe intensity zones for endurance training, scientific literature dealing with this topic in hypoxia is scarce, and some authors questioned the validity of these concepts at high attitude (Faulhaber et al. 2021). Lactate production [La] and clearance at rest and during exercise is influenced also by ambient but especially muscle temperatures, and magnitude and direction of this influence depend on the entity of cold (Therminarias 1992). Blomstrand et al. (1984) and No et al. (2016) suggested that higher levels of [La] are reached when muscle temperatures are low, due to a cold-induced change in muscle fibre recruitment from types 1 to 2 and a consequent greater reliance on anaerobic metabolism in this situation (Blomstrand & Essén‐Gustavsson, 1987), along with other factors contributing to fatigue, e.g., low levels of ATP and PCr (phosphocreatine). This would suggest that the net efficiency of exercise in the cold is lower than under normal conditions. However, Renberg et al. (2014) found no differences in blood lactate concentration at LT between − 14 and + 20 °C and Quirion et al. (1989) suggested that the anaerobic threshold corresponding to a lactate concentration of 4 mmol at − 20 °C is not significantly different compared to the threshold measured at + 20 °C.

Finally, also minute ventilation (Ve) is affected by acute hypoxic exposure, which results exaggerated compared to normoxia during exercise at a given absolute intensity. This allows arterial O_2_ (PaO_2_) to increase, despite the fact that the alveolar-to-arterial O_2_ pressure difference is increased during exercise (Calbet & Lundby 2009). However, this phenomena may be muffled or reversed when considering normobaric hypoxia (NH) due to greater air viscosity if compared to hypobaric conditions, especially at maximal exercise intensities (≈2.5% decrease in Ve_max_ per 1000 m of altitude gain in NH (Treml et al. 2020)). However, Friedmann et al. (2005) showed no differences in Ve at LT and Ofner et al. (2014) found similar ventilation in relative terms between normoxia and normobaric hypoxia. Also cold seems to have an influence on Ve (Oksa et al. 2004) since ventilating heavily cold air (< − 15 °C, Kennedy et al. 2020) may induce a bronchus constriction (induced by the contraction of bronchial smooth muscles), diminishing the amount of air that can be ventilated both at maximal and submaximal exercise intensities (Anderson and Daviskas 2000).

In the complex situation of combined cold and hypoxic environments, the effect of one stressor on performance, physiological and perceptual adjustments may be subject to change, simply due to the presence of the other independent stressor. Such differential influences can occur in three basic forms: additive, antagonistic, and synergistic (Lloyd and Havenith 2016), and each term defines a fundamental concept of inter-parameter interactions. Thus, the aim of this study is to provide further information regarding maximal, submaximal and lactate threshold responses when exposed to cold and hypoxic conditions, both independently and combined, taking into account the multifactorial approach proposed by Lloyd et al. (Lloyd and Havenith 2016). This should be helpful in better understanding the characteristics of interactions as well as their role in the operation of dynamic systems. On the basis of previous research (Lloyd et al. 2015, 2016), it was hypothesized that combined environmental stressor exposure will induce additive rather than synergistic effects on several physiological and perceptual parameters.

## Materials and methods

### Subjects

Fourteen trained (De Pauw et al. 2013) male subjects volunteered for this study (age: 27.3 ± 3.4, $\mathop {\text{V}}\limits^{.}$O_2max_: 64 ± 5.2 mL/min/kg, BMI:22.4 ± 1.7 kg/m^2^). All subjects were non-smokers, free of any systemic or chronic illness, and not taking medications. They were asked to refrain from intense physical activity on the day before and from drinking any alcohol and caffeinated beverages the day of the test. Furthermore, a nutrition diary was provided for writing down the meals of the day before and of the actual day of the first visit, to replicate as much as possible those meals also previous to the following sessions.

Thirteen subjects completed all experimental sessions, whereas one subject completed 4 out of 5 sessions. All study protocols were approved by the local ethics Committee (University of Verona- Project N. 4105CESC) and conformed to the Declaration of Helsinki. Before data collection, all participants were properly informed about the experimental procedures and gave their written informed consent for the measurements.

### Study design

Each participant visited the laboratory on 5 different occasions (once pre-test and four main tests) within the same time of the day and completed the protocol within a 6-week period. The pre-test defined subjects’ $\mathop {\text{V}}\limits^{.}$O_2max_ and individual Maximal Ascensional Velocity (VAM) through an incremental test to exhaustion on a motorized treadmill (slope: 25%, starting speed 2.0 km/h increased by 0.7 km/h every 3 min). Cardiorespiratory measures were collected continuously with breath-by-breath method using an automated open-circuit gas analysis system (Quark PFT Ergo, Cosmed Srl, Rome, Italy) and HR was recorded continuously during the test by a HR monitor incorporated into the gas analysis system. The results were used to define individual running speed in the exercise protocols for the four main tests.

The main tests were randomly performed in an environmental chamber in one of the following conditions: Normothermic Normoxia (N: 18 °C, 20.9% FiO_2_), Normothermic Hypoxia (H; 18 °C, 13.5% FiO_2_), Cold Normoxia (C: − 20 °C, 20.9% FiO_2_) and Cold Hypoxia (CH: − 20 °C, 13.5% FiO_2_). The hypoxic environment was created through the manipulation of the FiO2 by means of an oxygen dilution system based on the Vacuum Pressure Swing Adsorption principle (B-Cat, Tiel, The Netherlands). FiO2 was set at 13.5% to simulate an altitude ≈3500 m a.s.l. Each session consisted of a 30-min resting period already exposed to the specific environmental condition, followed by a 10-min warm up phase (2 km/h, slope 25%) and a submaximal to maximal test of 4-min intervals at increasing velocities, interspersed by 2 min of passive recovery performed in standing position on the treadmill using handrail support. The 30-min resting period is necessary to ensure that first short-term physiological responses to the hypoxic environment occur (Duffin 2007), but was repeated within all conditions to guarantee subject’s blindness to the experimental session. For the submaximal to maximal test, treadmill inclination was kept constant at 25% (Fornasiero et al. 2018) whereas test’s speed started from 30% of individual VAM (measured at pretest) and increased by 10% every interval until exhaustion. During cold conditions, participants wore extreme cold weather technical clothing individually chosen by each subject (including winter sport jacket/sweater, trousers, gloves and hat or band, with the only instruction of not covering the mouth with any scarf or neck warmer; estimated clothing insulation in the cold: 1.50 l_cl_(clo)), which remained identical for C and CH trials. Moreover, during exercise subjects were allowed to undress and during resting periods to wear additional clothing if they started to feel uncomfortable with their own clothes. Since in real life situations people generally adjust their clothing in order to not feel hot or cold, with this study we aimed at being as much ecological as possible letting the subjects choose their own clothes throughout the test: as a consequence we expect that, if any impact of cold on exercise performance and physiological parameters is present, it might be primarily related to airways limitations rather than to core temperature changes.

Throughout rest, exercise, and recovery phases, beat-to beat heart rate (HR) was continuously recorded using a Polar RS800CX HR monitor (Polar, Kempele, Finland). Pulse oxygen saturation (SpO2) was continuously recorded during exercise by ear pulse oximetry (Nonin Medical, Minneapolis, MN) at a sampling frequency of 1.0 Hz. Due to the extreme cold conditions, it was not possible to collect cardio-respiratory measures through the automated open-circuit gas analysis system. However, during resting conditions and the last 40 s of each exercise intensity (when steady state of V̇O_2_ was assumed to be reached), ventilatory data were collected using a flowmeter connected to a measuring system build on purpose for this project from our engineers. The flowmeter used was that of the Quark PFT system and it was calibrated with a 3-L syringe following exactly the instructions of the open-circuit gas analysis system.

The individual RPE was assessed using the CR100 Scale at the end of each exercise intensity (Borg and Borg 2002), together with thermal sensation (TS) using a 9-point scale (from -4 [very cold] to + 4 [very hot]) (Arens et al. 2006). To measure blood lactate, a blood sample was collected from the earlobe after the first minute of recovery at the end of each exercise intensity and 3, 5 and 7 min after test conclusion. The lactate analyser (Biosen C-line, EKF Diagnostics GmbH, Barleben, Germany) was calibrated according to the manufacturer’s instructions.

### Data analysis

Maximal workload (WL_max_) achieved at athlete’s exhaustion during all incremental tests was determined according to the following equation: WL_max_(km/h) = speed last stage completed (km/h) + [t(s)/step duration(s) * step increment(km/h)], where t is the time of the uncompleted stage (Kuipers et al. 1985).

Lactate thresholds were determined throughout three different descriptors (4mMol, Dmax and DmaxMOD (Fabre et al. 2010) thanks to the customised Lactate-E-excel worksheet (Newell et al. 2007): DmaxMOD results were finally chosen for discussion to overcome Dmax method underestimation of HR and to reduce the problems related to individual variability when considering fixed lactate concentration method (4mMol) instead of individual lactate kinetics for each subject (Fabre et al. 2010)**.** The DmaxMOD is a modified Dmax method, identified as the point on the third order polynomial curve that yielded the maximal perpendicular distance to the straight line formed by the point preceding an increase of lactate concentration greater than 0.4 mmol/L and the final lactate point (Bishop et al. 1998).

Ventilatory data were processed and analyzed with MATLAB 7.0 (The MathWorks, Inc., Natick, MA, USA), using a customized code to calculate minute ventilation (Ve), respiratory frequency (Rf) and tidal volume (Vt). Mean values of HR, Ve, Rf, Vt and SpO2 were averaged over the last 40 s of each submaximal exercise intensity until 80% of individual VAM (the last point in which we had data for all our subjects also in H and CH conditions). For maximal data, peak values registered during the last or the last but one stages were considered (since in some cases subjects completed less than 2 min during the last stage and given the 2-min recovery phase between stages, at the end of the test some parameters were still rising).

### Statistical analysis

Values presented are expressed as mean ± standard deviations (SD). All the data were tested for their normal distribution (Shapiro–Wilk test). When normality was not met, data were log transformed. [La], Thermal Sensation (TS) and SpO2 values at maximal level and WL, HR, RPE and ventilatory data at both maximal and threshold level were compared using a two-way ANOVA for repeated measures (RM), with ‘‘temp’’ (+ 18 °C and − 20 °C) and ‘‘Fiand’’ (normoxia and hypoxia) as factors. When an interaction effect (temp*FiO_2_) was found, Sidak post hoc test was used for specific comparisons (Cunha et al. 2015). Moreover, [La], HR, RPE, TS, SpO2 and ventilatory data at each submaximal exercise intensity until 80%VAM were compared using a three-way RM ANOVA, with ‘‘temp’’ (+ 18 °C and − 20 °C), ‘‘FiO_2_’’ (normoxia and hypoxia) and ‘’intensity’’ (INT30%, INT40%, INT50%, INT60%, INT70%, INT80%) as factors. When ‘temp*FiO_2_’ or ‘temp*FiO_2_*intensity’ interaction effects were found, Sidak post hoc test was used for specific comparisons (Cunha et al. 2015). Partial eta squared was calculated for each factor, individually and combined. Statistical analysis was completed using a statistical software (SPSS Inc, Chicago, Illinois, USA). The level of statistical significance was set at *p* < 0.05. Interpretation of partial eta squared values was conducted as follows: η^2^ < 0.01: negligible effect; 0.01 ≤ η^2^ < 0.06: small effect; 0.06 ≤ η^2^ < 0.14: moderate effect; η^2^ ≥ 0.14: large effect**.**

## Results

### Maximal WL, physiological and perceptual parameters

**Fig. 1:**
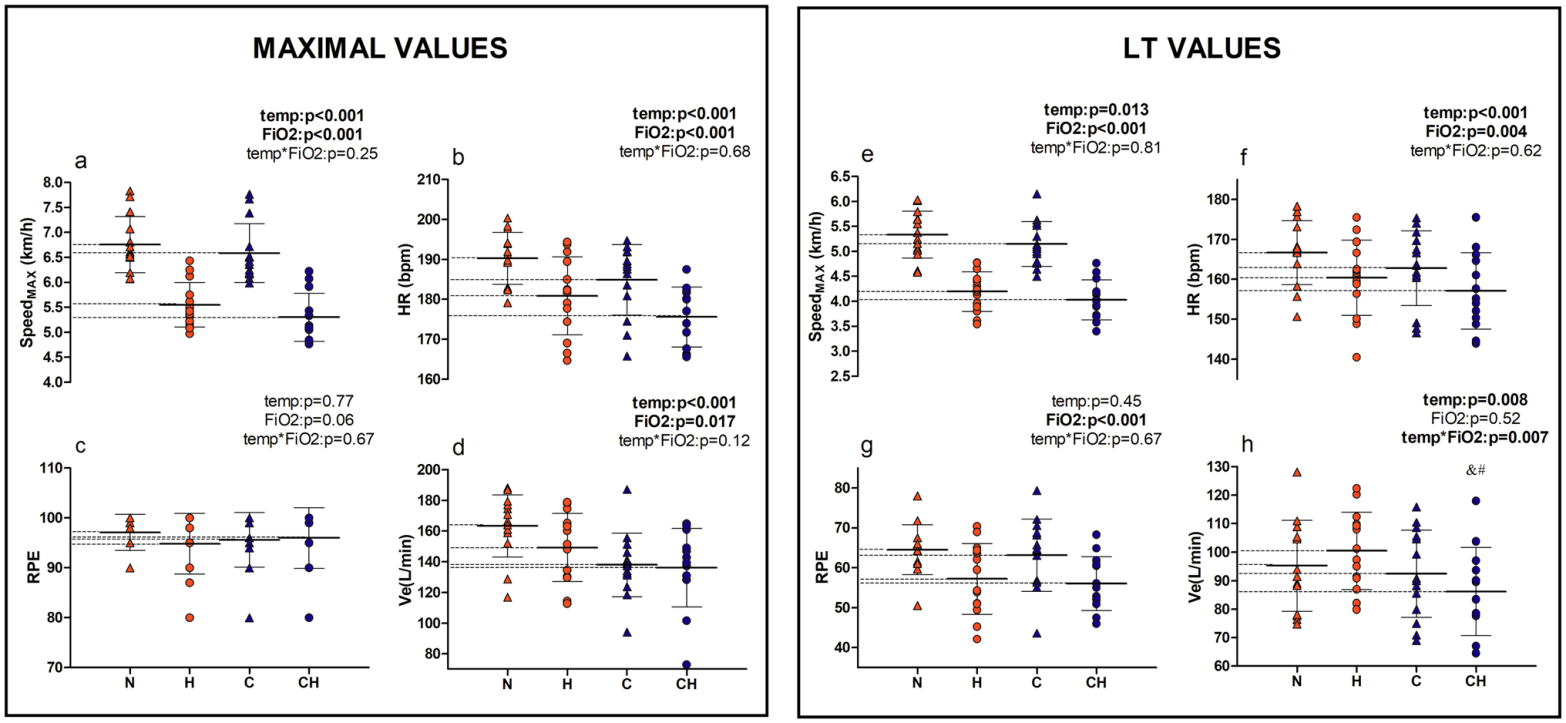
WL, HR, RPE and Ve at maximal (MAX) and lactate threshold (LT) intensities. N normothermic normoxia, red triangles; H normothermic hypoxia, red circles; C cold normoxia, blue triangles; CH cold-hypoxia, blue circles. Symbols indicate individual values, dark continuous line represents the mean. Dotted lines underline the differences between the means. &: CH ≠ C; #: CH ≠ H

Complete results from two-way RM ANOVA are reported in Table 1. [La] and RPE at maximal exercise were not different between environmental conditions. Main effects of hypoxia and cold were found for HR_max_, WL_max_, Ve_max_ and Vt_max_, which resulted lower in H and C compared to N, with no further significant reduction in CH. Oppositely, no individual but ‘temp*FiO_2_’ interaction effect was seen on Rf_max_, which was significantly higher in CH than in C and H alone. SpO2min was lower in hypoxic conditions, with no effect of cold. Finally, an interaction ‘temp*FiO_2_’ effect was seen in TS_max_, that was lower in the cold if compared to normothermic conditions, but was further reduced in CH if compared to C alone. Visual representation of maximal WL (Fig. 1a), HR (Fig. 1b), RPE (Fig. 1c) and Ve (Fig. 1d) in the four environmental conditions is reported below.

**Table 1:** Maximal values registered in the four experimental conditions

Maximal values														
		N	H	C	CH	FiO_2_	η^2^		Temp	η^2^		Inter	η^2^	
		Mean Sd	Mean Sd	Mean Sd	Mean Sd									
La	mMol	11.6 ± 1.9	11.6 ± 2.6	11.4 ± 2.1	11.8 ± 2.6	0.71	0.01	Small	0.98	0.00	Neg	0.63	0.02	Small
WL	(km/h)	6.8 ± 0.6	5.5 ± 0.5	6.6 ± 0.6	5.3 ± 0.5	< 0.001*	0.98	Large	0.001*	0.70	Large	0.25	0.11	Mod
HR	bpm	190 ± 7	181 ± 10	184 ± 9	176 ± 8	< 0.001*	0.91	Large	0.001*	0.83	Large	0.68	0.02	Small
Ve	L/min	163.3 ± 21	152 ± 20.6	139.5 ± 20.7	136.2 ± 25.6	0.017*	0.39	Large	< 0.001	0.71	Large	0.12	0.19	Large
Rf	bpm	64 ± 11	62 ± 11	61 ± 11	65 ± 13&#	0.53	0.03	Small	0.95	0.00	Neg	0.013*	0.41	Large
Vt	L/min	2.82 ± 0.4	2.6 ± 0.5	2.6 ± 0.6	2.3 ± 0.5	0.002*	0.54	Large	< 0.001*	0.88	Large	0.63	0.04	Small
RPE		97.7 ± 3.1	96 ± 4.5	96.8 ± 3.2	96.0 ± 6.1	0.06	0.26	Large	0.77	0.01	Neg	0.67	0.02	Small
TS		2.9 ± 1.1	2.8 ± 1.1	1.3 ± 1.4$	0.3 ± 1.5&#	0.007*	0.47	Large	< 0.001*	0.89	Large	0.047*	0.29	Large
SpO2	%	94.4 ± 2.9	75.1 ± 3.5	93.6 ± 5.1	76.3 ± 5.2	< 0.001*	0.95	Large	0.78	0.01	Neg	0.20	0.13	Mod

Maximal values for: [*La*] lactate, *WL* workload, *HR* heart rate, *Ve* ventilation, *Rf* respiratory frequency, *Vt* tidal volume, *RPE* rate of perceived exertion, TS Thermal Sensation, *SpO*_2_ minimum Pulse Oxygen Saturation at the end of exercise. *N* Normothermic Normoxia (18 °C, 20.9% FiO_2_), *H* Normothermic Hypoxia (18 °C, 13.5% FiO_2_), *C* Cold Normoxia ( − 20 °C, 20.9% FiO_2_) and *CH* Cold Hypoxia (− 20 °C, 13.5% FiO_2_). FiO_2_ general effect of fraction of inspired oxygen; temp: general effect of ambient temperature; inter: ‘temp*FiO_2_’ interaction effect. When a ‘temp*FiO_2_’ interaction effect was found, it has been reported as follow: $ C ≠ N, & CH ≠ C, # CH ≠ H. *p < 0.05. η^2^ < 0.01 negligible effect (neg), 0.01 ≤ η^2^ < 0.06 small effect, 0.06 ≤ η^2^ < 0.14 moderate effect (mod), η^2^ ≥ 0.14 large effect

### [La], WL, HR, RPE and ventilatory data at the lactate threshold

Mean and SD of [La], WL, HR, RPE and ventilatory data detected with the DmaxMOD method are presented in Table 2. WL and HR at threshold intensity were reduced both by cold and hypoxia, with no ‘temp*FiO2’ interaction effect, whereas RPE was reduced by hypoxia alone, with no effect of cold nor interaction. Also, HR and WL expressed as a percentage of maximal values in the respective conditions showed differences between normoxia and hypoxia. There were no differences in [La] concentration at LT in the four conditions. Vt showed a general decreasing effect of both cold temperature and FiO_2_ without interaction, whereas ‘temp*FiO_2_’ interaction was found for Ve and Rf, showing lower Ve in CH than C and H alone, but higher Rf than N only in H. Visual representation of WL (Fig. 1e), HR (Fig. 1f), RPE (Fig. 1g) and Ve (Fig. 1h) at LT in the four environmental conditions is reported below.

**Table 2:** Threshold values identified through the DmaxMOD in the four environmental conditions

Threshold values														
		N	H	C	CH	FiO_2_	η^2^		Temp	η^2^		Inter	η^2^	
		Mean Sd	Mean Sd	Mean Sd	Mean Sd									
La	mMol	4.1 ± 0.5	4.2 ±1.0	3.8 ± 0.5	4.2 ± 0.8	0.24	0.11	Mod	0.47	0.04	Small	0.39	0.06	Small
WL	km/h	5.4 ± 0.5	4.2 ± 0.4	5.2 ± 0.5	4.0 ± 0.4	< 0.001*	0.96	Large	0.013*	0.42	Large	0.81	0.01	Neg
HR	bpm	167 ± 8	160 ± 10	162 ± 9	157 ± 10	0.004*	0.51	Large	< 0.001*	0.66	Large	0.62	0.02	Small
VE	L/min	97 ± 15	102 ± 13	94 ± 15	86 ± 15&#	0.52	0.04	Small	0.008*	0.46	Large	0.007*	0.47	Large
Rf	bpm	40 ± 6	46 ± 8§	43 ± 7	45 ± 9	0.014*	0.41	Large	0.47	0.05	Small	0.032*	0.33	Large
Vt	L/min	2.5 ± 0.4	2.3 ± 0.5	2.2 ± 0.5	2.0 ± 0.5	0.007	0.47	Large	< 0.001	0.80	Large	0.389	0.06	Mod
RPE		66 ± 5	58 ± 8	65 ± 7	56 ± 7	< 0.001*	0.74	Large	0.45	0.05	Small	0.67	0.02	Small
WL%max	%	79 ± 4	76 ± 4	78 ± 3	76 ± 4	0.006*	0.48	Large	0.77	0.01	Neg	0.49	0.04	Small
HR%max	%	83 ± 2	87 ± 2	84 ± 2	88 ± 2	< 0.001	0.85	Large	0.06	0.27	Large	0.60	0.02	Small

*DmaxMOD* modified Dmax method for LT determination, [*La*] blood lactate WL Workload, *HR* heart rate, Ve ventilation, *Rf* respiratory frequency, *Vt* tidal volume, RPE rate of perceived exertion, *WL*%max WL expressed as a percentage of maximal values in the respective conditions, *HR*% HR expressed as a percentage of maximal values in the respective conditions. *N* Normothermic Normoxia (18 °C, 20.9% FiO_2_), *H* Normothermic Hypoxia (18 °C, 13.5% FiO_2_), *C* Cold Normoxia ( − 20 °C, 20.9% FiO_2_) and CH: Cold Hypoxia (− 20 °C, 13.5% FiO_2_). FiO_2_: general effect of fraction of inspired oxygen; temp: general effect of ambient temperature; inter: ‘temp*FiO_2_’ interaction effect. When a ‘temp*FiO_2_’ interaction effect was found, it has been reported as follow: §: H ≠ N; &: CH ≠ C; #: CH ≠ H. *p < 0.05. η^2^ < 0.01: negligible effect (neg); 0.01 ≤ η^2^ < 0.06: small effect; 0.06 ≤ η^2^ < 0.14: moderate effect (mod); η^2^ ≥ 0.14: large effect

### Submaximal physiological and perceptual adjustments

**Fig. 2:**
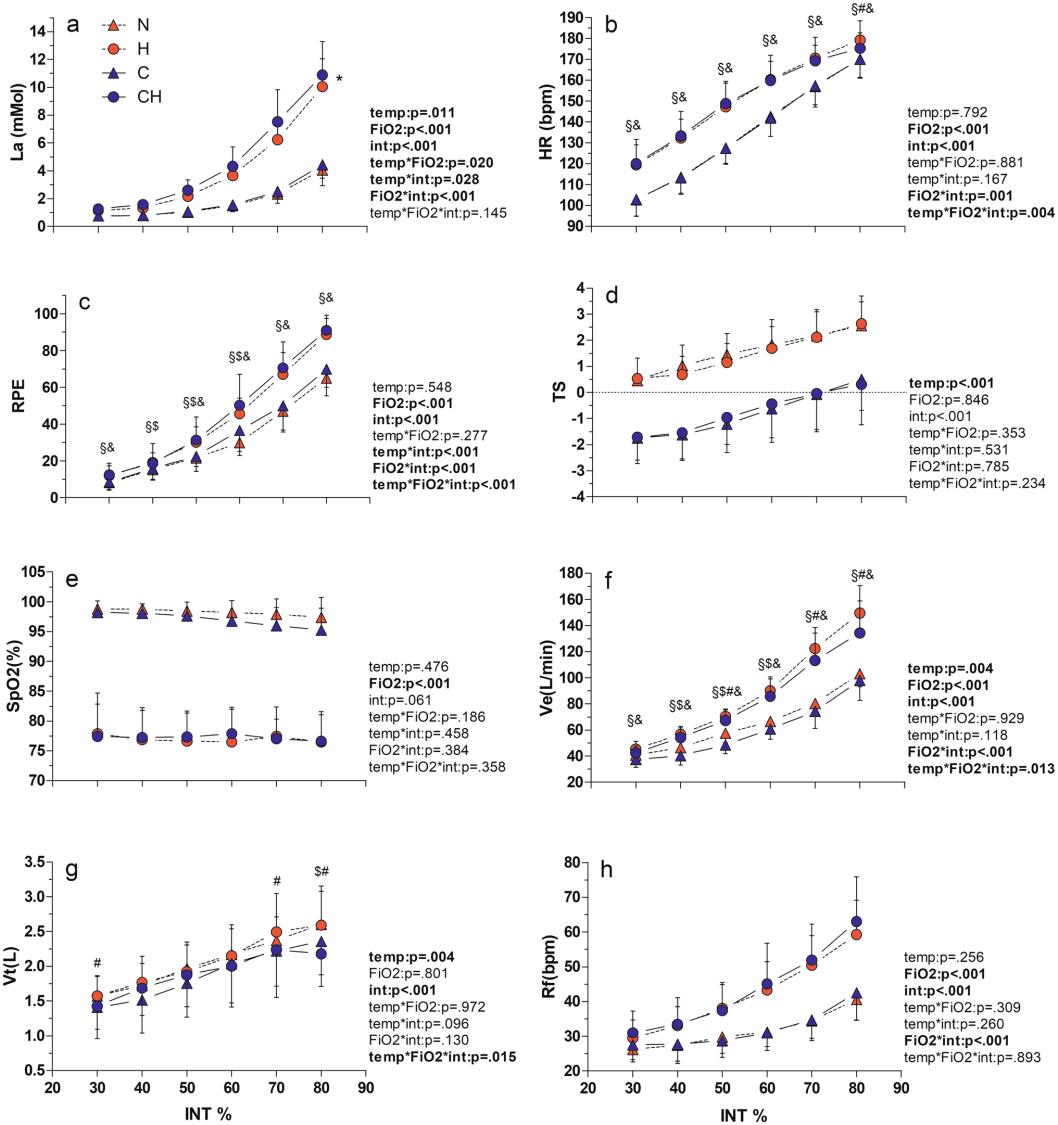
Mean submaximal values in the four environmental conditions. Lactate [La], Heart Rate (HR), RPE, Thermal Sensation (TS), Pulse Oxygen Saturation (SpO_2_), Ventilation (Ve), Tidal Volume (Vt), Respiratory Frequency (Rf) during an incremental treadmill test performed in normothermic normoxia (N, red triangles), normothermic hypoxia (H, FiO_2_ 13.5%;red circles), cold normoxia (C,−  20 °C; blue triangles) and cold-hypoxia (CH, blue circles). Data are reported until 80% of individual maximal ascensional velocity. Values are presented as means ± standard deviations. When a ‘temp*FiO_2_*intensity’ interaction effect was found, it has been reported as follows: §H ≠ N; $C ≠ N; &CH ≠ C; #CH ≠ H. When a ‘temp*FiO2’ interaction effect was found, it has been reported as follow: * overall CH curve ≠ overall H curve

Mean and SD data for submaximal exercise intensities as well as complete results from three-way RM ANOVA are reported in Fig. 2. Given the nature of a maximal incremental test to exhaustion, a general effect of ‘intensity’ was detected for each of the examined variables except for SpO2 **(**Fig. 2e**)**. [La] accumulation (Fig. 2A) was generally increased by both cold and hypoxia, showing a ‘temp*FiO_2_’ but not a ‘temp*FiO_2_*intensity’ interaction: post hoc comparison showed that overall [La] curve was left-shifted only in CH if compared to H alone, but not in C if compared to N. HR (Fig. 2b) was always higher in the hypoxic conditions, but at INT80 it was also higher in H if compared to CH condition (‘temp*FiO_2_*intensity’ interaction effect). RPE (Fig. 2c) showed a general effect of hypoxia but also a ‘temp*FiO_2_*intensity’ interaction: post hoc comparison revealed that in H and CH subjects had higher RPE than temperature matched normoxic conditions (N and C, respectively) for each exercise intensity, but also that RPE at INT40, INT50 and INT60 was higher in C if compared to N. TS (Fig. 2d) was lower in the cold regardless of present FiO_2_ at each 4-min submaximal exercise intensity. As for maximal values, SpO2 during submaximal exercise in normobaric hypoxia was significantly lower than in normoxia, with no further effect of environmental temperature or exercise intensity (Fig. 2e). General effects of hypoxia and cold were seen on ventilatory parameters: Ve and Rf (Fig. 2f and Fig. 2h) were higher in the hypoxic conditions if compared to the normoxic ones, whereas a general effect of cold on Ve (Fig. 2f) and Vt (Fig. 2g) was found. A ‘temp*FiO_2_*intensity’ interaction effect on Ve revealed that this parameter decreases significantly at high exercise intensities in the cold only when also hypoxia is present (Ve in CH is lower than in H alone at INT70 and 80, but Ve in C is similar to N).

## Discussion

Even though the independent effects of cold and hypoxia on performance and physiological and perceptual adjustments to exercise have been widely described in literature (Castellani and Tipton 2016; Robert S.Mazzeo 2006), their combined influence on these aspects have been only investigated on segmental exercising tasks (i.e., Knee extensors and finger flexors); (Lloyd et al. 2015, 2016)). Indeed, to the best of our knowledge, this is the first study examining whole body endurance exercise responses when combinedly exposed to hypoxia and cold ambient temperature. The key findings of this study were that performance (WL_max_ and WL at LT) and most physiological variables (HR_max_ and HR at LT, Ve_max_ and Vt) show an additive rather than interactive effect (Lloyd and Havenith 2016) in the CH condition, i.e., the decrease of above-mentioned variables by the combination of cold and hypoxia was equal to the sum of the effects exerted by the two environmental stressors alone. [La] levels throughout the test displayed an overall synergistic effect of cold and hypoxia on this parameter. Moreover, Ve at LT was characterized by synergistic effect of the two conditions, decreasing significantly in CH. Finally, RPE at lactate threshold intensity is exclusively reduced by hypoxia (i.e., exclusive effect), whereas TS_max_ is synergistically reduced by cold and hypoxia.

### Maximal and LT workload

It is already well documented that exercise performance for endurance-type efforts deteriorates in hypoxia (Amann et al. 2006; Doubt 1991; Fornasiero et al. 2018; Goodall et al. 2022), whereas it is still controversial if this occurs in response to a cold stimulus (Castellani and Tipton 2016; Castellani and Young 2016), primarily due to different tested ambient temperatures. In our study, we found a 18% reduction in maximal exercise capacity in H, higher than the 10 to 13% decrease previously found for altitudes between 2500 and 3500 m (Faulhaber et al. 2021; Friedmann et al. 2004, 2005; Lorenz et al. 2006; Ofner et al. 2014; Weckbach et al. 2019). However, WL reduction (-21%) at LT in H is in line with previous defined reduction in PO at LT in hypoxia (≈11 to 19% Faulhaber et al. 2021; Weckbach et al. 2019)), especially if considering greater simulated altitude in this study if compared to the others (3500 vs 3000 and 2650 m asl). In C, our data show a 2.3% reduction in WL_max_, much lower than the 20% decrease found by Quirion et al. (1989) at − 20 °C. Also WL at LT is decreased in C (-3.5%): previous research demonstrated greater WL at LT within the so defined optimal ambient temperature (from − 4 to 0 °C, if properly dressed Morrissey et al. 2019; Sandsund et al. 2012)) if compared to higher or lower ambient temperatures. On the other hand, others claim no differences in WL at LT or VT between + 20 °C and − 14 °C (Renberg et al. 2014; Therminarias 1992; Therminarias et al. 1989)**.** Our results partially disagree with these findings, and this may be due to the much lower ambient temperature tested in this study.

A novel finding was that combining cold and hypoxia induced an additive rather than a synergistic effect (Lloyd and Havenith 2016), further compromising WL at maximal (− 21.5%) and LT level (− 24.6%) in CH. These observations pertain to workload, as we did not have the possibility to measure actual V̇O_2_ due to constraints in the use of measurement tools in the cold. If on one hand the main cause for WL_max_ reduction in H is linked to $\mathop {\text{V}}\limits^{.}$O_2max_ reduction (Wehrlin and Hallén, 2006), on the other hand, maximal exercise capacity in the cold might be impaired by both $\mathop {\text{V}}\limits^{.}$O_2max_ reduction (Oksa et al. 2004; Quirion et al. 1989), as well as by cold induced bronchoconstriction and reduced mechanical efficiency (Castellani and Tipton 2016; Sandsund et al. 2012). In fact, muscle cooling impairs most functional properties, including a reduction in both the shortening and lengthening velocity of the muscle and in the capacity of power expression in agonist muscle groups (Renberg et al. 2014; Wiggen et al. 2013). The reason for additive and not synergistic effect on WL in CH may be explained as follows: firstly, evidences in exercise capacity impairments in the cold are not clear and thus the low impact magnitude of this stressor on performance is what may dictate the ‘additive’ and not ‘synergistic’ type of interaction expressed between these two stressors (Lloyd et al. 2016). Moreover, the physiological mechanisms leading to WL changes in hypoxic and cold environments could not always share a common pathway of action (PaO2 reduction in hypoxia vs bronchoconstriction and reduced mechanical efficiency in the cold), and as proposed by previous authors, interactive effects probably arise only when combining stressor that are mechanistically similar (Broadbent 1963; Lloyd et al. 2016). In the opinion of the authors, since our subjects were well dressed and did not feel cold at the end of exercise, muscle temperature and blood flow were probably preserved, remaining bronchoconstriction the principal cold-induced effect that led to WLmax reductions in our cold trials; for this reason, the ‘mild stressor effect’ of cold remains the preferred explanation for additive rather than interactive effects between our stressors.

### Maximal and submaximal physiological responses

#### Heart rate

Similarly to previous published data (Fornasiero et al. 2018; Grataloup et al. 2007; Mourot 2018; Ofner et al. 2014), we showed a decrease in HR_max_ when acutely exposed to H and this reduction (− 10 bpm at 3500 m) appears to be slightly higher than the proposed average decrease of 1.7 bpm per 1000 m gain in altitude (Garvican–Lewis et al. 2015). However, Mollard et al. (Mollard et al. 2007) demonstrated that at 3500 m of altitude HR decreased by 11 bpm in trained ($\mathop {\text{V}}\limits^{.}$O_2max_ > 60 ml/kg/min, as for our subjects) and by 5 bpm in untrained ($\mathop {\text{V}}\limits^{.}$O_2max_ < 50 ml/kg/min) subjects, proving an effect of training status on HR reduction in hypoxia (Richalet R et al. 1992; Richalet 1988). The explanations for the reduction in HR_max_ when acutely exposed to hypoxia include (i) a change in cardiac electrophysiological properties (i.e., increased duration of repolarization length and slower atrio-ventricular conduction) (Benoit et al. 2003; Grataloup et al. 2007; Mourot 2018), (ii) a decrease in muscle $\mathop {\text{V}}\limits^{.}$O_2max_ due to arterial hypoxemia that leads to reduced cardiac output (CO) (Benoit et al. 2003) and (iii) a decrease in exercise effort due to reduced oxygen content that is perceived by the central nervous system, causing accelerated development of muscle fatigue: this implies an increase in inhibitory afferent signals and a reduced central drive on the heart as a protective mechanism from myocardial ischemia (Noakes et al. 2001).

More debated is the topic on HR at LT in hypoxia: it is often assumed that training with the same HR in normoxia and hypoxia would result in equivalent training intensities in these environments (Brosnan et al. 2000; Ofner et al. 2014). However, Friedmann et al. (2005;2004) showed a reduction in HR at LT (detected with different methods) ranging from − 3 to 4% at 2500 m if compared to sea level values: similarly, we found a 3.8% reduction in HR at LT at 3500 for our subjects, suggesting that HR reductions at LT in H may be characterized by a ceiling effect as altitude increases. Interestingly, as opposed to Friedmann et al. (2004), also HR expressed as a percentage of maximal values in the respective conditions showed differences between normoxia and hypoxia. This point is of paramount importance for training prescription to prevent overtraining, for performance to target best pacing strategy, but also for the design of scientific studies to guarantee same relative exercise intensity in studies confronting normoxic and hypoxic environments.

We also found a general effect of cold on maximal HR, which decreased on average by 3.2% in C if compared to N, in line to the ≈4% decrease that has been found for ambient temperatures between − 14 and − 20 °C if compared to + 20 °C (Oksa et al. 2004; Renberg et al. 2014; Sandsund et al. 2012). No effect of cold on submaximal HR when considering same external workload was found, but HR at LT was reduced by 2.8% in C. Our result disagrees with Therminaris et al. 1989, who determined that HR at − 2 °C was reduced up to moderate exercise intensities, remaining unchanged at LT: the magnitude of cold (− 20 vs − 2 °C) could be addressed as a possible explanation for these differences. The reasons for HR reductions in the cold are still controversial but have been mainly related to cold induced peripheral vasoconstriction that increases central blood volume and consequently stroke volume (Doubt 1991; Gisolfi and Wenger 1984), implying a reduced sympathetic drive and a change in heart mechanics (Castellani and Tipton 2016; Doubt 1991; Sandsund et al. 2012). However, also lower external work performed both at maximal and LT intensities could play a major role in this reduction (Castellani and Tipton 2016).

The CH condition induced an additive decreasing effect (Lloyd and Havenith 2016) of C and H on HR_max_ (H: − 5.0%, C: − 3.2%, CH − 7.7% if compared to N, respectively) and a relative additive effect on HR at LT ( H:− 3.81%, C:− 2.80%, CH:− 5.69%). A relative additive effect displays a situation in which the combination effect of two stressors on one variable is lower than the sum of independent effect, but to an extent that do not induce a real antagonistic effect between the 2 stressors: this is probably due to the fact that C and H alone can be considered as mild stressors that operate on the heart with partial independent mechanisms (see above) (Broadbent 1963; Lloyd et al. 2015, 2016) showing just a tendency towards the ‘worst strain take precedence principle’ on exercising HR in CH.

#### Lactate

There were no differences in [La]_max_ between environmental conditions, suggesting that our subjects reached exhaustion in all conditions despite reduced WL_max_ in H, C and CH. [La] was significantly higher in hypoxic environments from the beginning to the highest exercise intensities (Fig. 2a). This result was expectable (Clark et al. 2007; Friedmann et al. 2005), considering that the absolute workload of the protocol was the same for the 4 sessions. However, [La] at LT was not different between normoxic and hypoxic conditions (Table 2). No differences in [La] accumulation throughout the test (Fig. 2a) nor at LT intensity (Table 2) were seen between C and N conditions: the influence of cold exposure on blood lactate response has been debated in literature, suggesting that it depends on several factors, such as intensity and duration of cold exposure. Therminaris et al. (1989) found greater [La] levels below LT and lower [La] levels above LT at -2 °C ambient temperatures if compared to + 20 °C, and Blomstrand et al. (1984) suggested that most subjects at reduced muscle temperature attain higher muscle lactate concentrations for same exercising workload (Blomstrand et al. 1984), as well as show delayed but higher peak blood lactate concentration at the end of exercise (Blomstrand & Essén‐Gustavsson 1987), indicating a lower flux of lactate from muscle to blood in this condition. This increase in blood and muscle lactate concentration at low muscle temperatures suggests a greater reliance on anaerobic metabolism: cold exposure, as well as muscular exercise, stimulates the sympathoadrenal system, increasing plasma catecholamine concentrations that are responsible for increased muscle glycogenolysis (Himms–Hagen 1972). Moreover, reduced local blood flow could lead to decreased oxygen delivery during exercise (with consequently higher reliance on anaerobic metabolism) and delayed muscle lactate release from the muscle, further enhancing the accumulation of lactate in the muscle (Blomstrand & Essén‐Gustavsson 1987; Castellani and Tipton 2016). The reason why we did not find any difference in [La] accumulation throughout the test in C if compared to N may be related to the fact that our subjects were allowed to wear the clothes they preferred, thus never causing a decrease in muscle temperature to an extent that determined a change in lactate metabolism. More controversial are the results concerning hypoxic trials, in which [La] curve was left-shifted in CH if compared to H, suggesting an effect of cold on [La] metabolism only when combined to the hypoxic stressor. Furthermore, [La] at LT in CH was similar to the other conditions (Table 2), but corresponded to the lowest exercising WL (additive effect between C and H), confirming that in CH higher values of La are expected when considering same WL as both H or C. Further studies are needed to better clarify this aspect, accurately measuring muscle temperature, blood flow and PaO_2_ in cold-hypoxic environments.

#### Ventilation

Ve_max_ (H = − 6.9%, C = − 14.6%, CH = − 16.6%) and Vt_max_ (H:− 7.05%; C:− 9.57%, CH:− 18.65%) decreased from N with both a general effect of hypoxia (*p* = 0.017 and 0.002) and cold (both *p* < 0.01), displaying a partial (i.e., Ve) or complete (i.e., Vt) additive effect in CH without any statistical interaction. Conversely, Rf_max_ was similar to N in CH (despite the lowest WL_max_), but was significantly higher than in H (+ 5.7%) and C (+ 7.0%) alone.

Ve_max_ in normobaric hypoxia has been shown to be slightly decreased if compared to hypobaric hypoxic conditions (at comparable simulated altitude level) (Treml et al. 2020), probably due to distinct breathing patterns related to changes in air density that differently affect the central motor drive (Amann and Dempsey 2016). However, the lower Ve_max_ in hypoxia at reduced exercise performance in our study has to be emphasized. Conversely, ventilating heavily cold air induces bronchus constriction (Oksa et al. 2004), diminishing the amount of air that can be ventilated (Anderson and Daviskas 2000): in fact, the rapid recruitment of the smaller airways into the heating and humidifying process cause a quicker water loss, creating a greater osmotic gradient than that related to warm air breathing. Bronchoconstriction leads to the so-called dynamic hyperinflation mechanism: some air remains trapped in the lungs, causing a temporary increase in end expiratory lung volume above its baseline level, and reducing tidal volume and consequently ventilation, since not properly compensated by increased respiratory frequency (Stickland et al. 2022). Furthermore, Kennedy et al. (Kennedy et al. 2020) claim that exercise induced bronchoconstriction could be greater at temperature colder than − 15 °C if compared to 0 °C, which is exactly the case for the proposed study. Interestingly, in CH there is a complete additive reduction effect of H and C on Vt, but just a relative additive reduction effect on Ve, since probably Rf_max_ was increased to overcome cold induced Vt reductions. However, when considering same relative submaximal exercise intensity at LT, Ve is lower in CH than in H and C alone, remaining similar to N in the other experimental conditions (H:− 6%; C:− 3%; CH:− 11% if compared to N). This is linked to a complete additive effect of H (− 9%) and C (− 10%) on Vt in CH (− 19%) (as it happens at maximal level), combined to a nullification (Lloyd and Havenith 2016) effect of the 2 environments on Rf increase from N (H: + 16%, C: + 8%, CH: + 13%), possibly also related to reduced exercising WL in this condition. Finally, considering submaximal exercising WLs, both Vt and Ve (Fig. 2g and f) seem to be affected by the presence of cold. All these information confirm the effect of extreme cold on respiratory mechanics while exercising, but also the necessity to deepen knowledge on the interaction role of hypoxia and cold on Ve, Vt and Rf at both maximal and submaximal intensities. Further studies on ventilatory responses, cold induced bronchoconstriction and exercise performance in CH should be implemented.

### Maximal and submaximal perceptual reponses

#### RPE

No differences were seen in RPE_max_, further confirming (together with [La]_max_) that our subjects reached maximal effort in all experimental sessions. RPE at submaximal exercise was higher in hypoxic conditions (due to same absolute but different relative intensity), with no differences in CH if compared to H alone. More controversial is RPE reduction at LT in hypoxia: Aliverti et al. (2011) explained that both leg and breathing RPE are higher in hypoxia at same leg power output to normoxia (due to different relative exercise intensities), but breathing RPE is the same in normoxia and hypoxia when considering equal Respiratory muscles power output (PO) at similar ventilation rates. Interestingly, reduced RPE values at LT has been given by our subject in H, mainly due to decreased leg PO in this condition, but a further reduction in RPE was seen in CH, the only condition in which Ve at LT was lower than in the other experimental sessions. This confirms the idea that the lower the ventilatory response (and thus respiratory muscle PO), the lower the perceived exertion despite similar relative exercise intensity (Nicolò et al. 2015). The mechanisms underlying this phenomena should be further studied, especially considering the valuable importance of RPE as a monitoring tool for training when physiological parameters are not so reliable (i.e., at high altitude).

#### TS

At maximal exercise, TS in CH resulted lower than in C alone, implying an effect of hypoxia in the perception of cold that could be related to the reduced mechanical work performed at the end of exercise. In fact, TS at submaximal level (and same exercising WL) showed only an effect of ambient temperature, being lower in cold than normothermic conditions, with no further effect of hypoxia. The interaction effect of cold and hypoxia on the perception of thermal stimuli has been extensively debated in literature (Golja et al. 2004; Keramidas et al. 2019; Malanda et al. 2008; Massey et al. 2015), with the most quoted idea being that acute exposure to normobaric hypoxia in combination with whole body cooling results in vasoconstriction at warmer skin temperature compared to a normoxic condition (Massey et al. 2015); however, decreased neural processing and/or decreased nerve conduction speed in the sensor-to-effector pathway (Malanda et al. 2008) alters subjects thermal perception and comfort, consequently attenuating thermoregulatory behaviour during cold exposure at altitude (Golja et al. 2004). It is possible that at submaximal exercise intensities, our subjects perceived the same TS value in C and CH, even though in the latter condition the thermal stimulus for the body was worse. Also greater relative exercise intensity (and consequently heat production) in CH associated with same perceived TS as C alone underlines impaired thermal perception that requires further attention in future studies for both safety and performance reasons.

## Limitations

For this study, we could not record any metabolic data in the cold conditions due to the technical impossibility of using the breath-by-breath metabolimeter at temperatures below zero (manufacturer instructions temperature range: 10–40 °C). However, going deeper into exercise physiology in the cold is warranted, and future studies should consider the use of Douglas bags for Oxygen consumption measurements. Moreover, some results (i.e., ventilation) may have been affected by the fact that we studied acute exposure to normobaric hypoxia, which is known to induce some different physiological adjustments with respect to hypobaric exposure. In addition, we do not have any information regarding subjects core or skin temperature, which would have allowed us to exclude any role of reduced overall body’s temperature in measured outcomes. Finally, repeating the same incremental test in the four conditions causes lower exercise durations in hypoxia and this may also affect some results: a piece of advice for the future is the use of matched exercise intensity protocols between normoxic and hypoxic conditions, that should partially overcome this problem and allow easier comparison of measured variables between conditions.

## Conclusion and future perspective

The combination of cold (− 20 °C) and hypoxia (≈3500 m) exerted additive rather than synergistic effects on exercise performance, decreasing LT and maximal exercising workload to an extent that is equal to the sum of the two stimuli alone. Both exclusive effects of hypoxia (i.e., maximal and submaximal SpO, submaximal Rf and RPE at LT) and cold (i.e., submaximal Vt and TS) and different additive (i.e., maximal and LT HR, Ve _max_) and synergistic (i.e., TS_max_ and Ve at LT) effects of the two stressors were found on the investigated maximal and submaximal physiological and perceptual variables. Future studies should (i) better understand the magnitude of influence of cold induced bronchoconstriction on exercise performance, especially when combined to acute hypoxic ventilatory response and (ii) consider combined cold and hypoxic effect on performance when also a significant reduction in core and muscle temperature is expected, accurately measuring these parameters. These results provide new insight into human responses to exercise in cold and hypoxic environments, highlighting the need for careful consideration of independent and combined stressor impact on considered variables for optimal exercise intensity prescription and training load monitoring in athletes training/competing in hypoxic and/or cold environments.

## Data Availability

The data that support the findings of this study are available upon reasonable request from the corresponding author.

## Abbreviations

ANOVA: Analysis of Variance
C: Cold Normoxia
CO: Cardiac Output
CH: Cold-Hypoxia
H: Normothermic Hypoxia
HR: Heart Rate
LT: Lactate Threshold
[La]: Blood Lactate Concentration
N: Normothermic Normoxia
PaO2: Arterial O2 pressure
RPE: Rate of Perceived Exertion
TS: Thermal Sensation
Ve: Ventilation
$\mathop {\text{V}}\limits^{.}$O2max: Maximal oxygen consumption
WL: WorkLoad
